# Editorial: Autonomic regulation of cardiovascular function and implications for future therapeutic approaches

**DOI:** 10.3389/fphys.2022.1039876

**Published:** 2022-10-28

**Authors:** Bruce H. KenKnight, Richard L. Verrier

**Affiliations:** ^1^ LivaNova PLC, Houston, TX, United States; ^2^ Medicine/Beth Israel Deaconess Medical Center, Harvard Medical School, Boston, MA, United States

**Keywords:** autonomic regulation therapy, vagus nerve stimulation, heart failure, reduced left ventricular ejection fraction, T-wave alternans, heart rate variability

For more than a century, experimental and clinical studies have implicated a critical role of the autonomic nervous system in regulation of cardiovascular function in health and disease. In recent years, there has been increasing attention to the possibility of applying autonomic interventions to address the challenging problem of alleviating morbidity and reducing mortality in patients afflicted with heart failure (HF) with reduced left ventricular ejection fraction (HFrEF). This is an important public health problem throughout the world since more than 26 million people are affected (doi: 10.15420/cfr.2016:25:2), and, in the United States alone, HF is a major cause of death resulting in >377,000 deaths annually (doi: 10.1161/CIR.0000000000001052).

The prospect of applying chronic vagus nerve stimulation (VNS) to improve HF symptoms and status has been the focus of intensive study because of extensive preclinical and clinical evidence of its potential for cardioprotective actions. An attractive consideration of chronic VNS is its well-established safety profile and tolerability, determined in the treatment of >120,000 individuals with chronic epilepsy or depression, which has been under study for more than three decades. In this experience, it has been shown that chronic VNS in patients with pharmacoresistant epilepsy can safely reduce cardiac electrical instability and improve autonomic function.

The putative mechanistic bases for cardioprotection by chronic VNS as revealed by preclinical studies are multifold. Evidence of rich cholinergic innervation of the atrioventricular node and ventricular conducting system has been provided not only in widely studied canine models but also in human subjects. Multiple modes of action underlie the bases by which chronic VNS can prevent life-threatening arrhythmias including ventricular fibrillation. Among the most critical influences is antagonism of cardiac sympathetic nerve tone. Elegant studies of skin sympathetic nerve activity have shown that VNS is capable of containing adrenergic inputs to the heart (Chen et al.).

The articles in the current research topic reviewed the main candidate effects for the protective action of VNS in the treatment of heart failure. Among the most significant are anti-inflammatory actions involving VNS-mediated decreases in circulating cytokines and augmentation of nitric oxide expression (Allen et al.) and cardiac antiapoptotic effects. Improvement in autonomic balance as assessed by heart rate variability (HRV) and baroreceptor sensitivity (BRS) by heart rate turbulence measurement has been documented (Verrier et al.). Overall, this suite of influences has been shown to improve both cardiac mechanical function and cardiac electrical stability as assessed by measures such as T-wave alternans and T-wave heterogeneity.

Schwartz and colleagues (doi: 10.1016/j.ejheart.2008.07.016. PMID: 18760668) are to be credited for taking a critical step of conducting the first-in-man proof of concept investigation in patients with HF. Their study demonstrated that VNS improved HF symptoms and decreased left ventricular end systolic volumes. Importantly, the patients experienced relatively minor adverse effects of chronic VNS that tended to resolve over time, similarly to patients with epilepsy. Early clinical studies using VNS appeared promising; however, the larger INcrease Of VAgal TonE in Heart Failure (INOVATE-HF) trial and Neural Cardiac Therapy for Heart Failure Study (NECTAR-HF) were neutral with respect to the primary clinical endpoints. The only sizeable study with definitively favorable results has been the Autonomic Regulation Therapy to Enhance Myocardial Function and Reduce Progression of Heart Failure (ANTHEM-HF) Pilot study (Verrier et al.). The main apparent distinctive feature of ANTHEM-HF was use of the neural fulcrum to ensure appropriate titration of stimulation intensity that demonstrated efferent autonomic engagement in individual patients. This approach is reminiscent of the historical rationale used in pharmacologic therapy, specifically, “to administer the right drug, to the right patient, by the right route, at the right time, in the right amount, for the right duration” (doi: 10.1177/106002809002400615).

In support of the view that appropriate VNS intensity and protocol are essential is the fact that the ANTHEM-HF Pilot study resulted in multiple long-lasting benefits of chronic VNS on cardiac electrical stability and mechanical function in patients with HFrEF for more than 3 years. Specifically, there were progressive improvements in HRV, BRS, cardiac electrical stability as assessed by T-wave alternans and T-wave heterogeneity, and reduction in incidence of nonsustained ventricular tachycardia. Concurrently, there were significant improvements in left ventricular ejection fraction (LVEF), 6-min walk tests, and NYHA Class. It is noteworthy that the NYHA Class was improved in 96% of subjects. Furthermore, durable cardioprotective effects in terms of autonomic engagement and heart rate responsiveness to VNS were also observed in 15 patients enrolled in the ANTHEM-HF Pilot Study for an additional 2 years (Libbus et al.).

Based on the sizeable body of evidence provided by prior preclinical and clinical studies, the Autonomic Regulation Therapy to Enhance Myocardial Function and Reduce Progression of Heart Failure with Reduced Ejection Fraction (ANTHEM-HFrEF) Pivotal Study (NCT03425422) (LivaNova PLC, Houston, TX, United States) has been launched (Konstam et al.). The projected enrollment is 500 to 1,000 patients. The study design utilizes an adaptive, open-label, randomized controlled protocol. This investigation draws on insights derived from prior studies that optimized VNS dose, stimulation parameters, and selection of appropriate patients, together with a novel adaptive approach for guiding sample size, as allowed by the new United States Food and Drug Administration *Breakthrough Device Program*.

The development period of neuromodulation approaches to the management of HF appears to have been lengthy. However, as pointed out by Byku and Mann (2016; doi: 10.1016/j.jacbts.2016.03.004), “it is instructive to recall that cardiac resynchronization therapy, which is now a class I indication in HF patients, took over two decades to evolve from a concept in animal models to widespread clinical application.” The ANTHEM-HFrEF Pivotal Study could accelerate this timetable, given that the first-in-man study of VNS for HF was published in 2008.

